# SOAT1 Promotes Gastric Cancer Lymph Node Metastasis Through Lipid Synthesis

**DOI:** 10.3389/fphar.2021.769647

**Published:** 2021-11-01

**Authors:** Tingting Zhu, Zhangding Wang, Tianhui Zou, Lei Xu, Shu Zhang, Yali Chen, Chen Chen, Weijie Zhang, Shouyu Wang, Qingqing Ding, Guifang Xu

**Affiliations:** ^1^ Department of Gastroenterology, The Affiliated Drum Tower Hospital of Nanjing University Medical School, Nanjing, China; ^2^ Division of Gastroenterology and Hepatology, Renji Hospital, Shanghai Jiao-Tong University School of Medicine, Shanghai Institute of Digestive Disease, Key Laboratory of Gastroenterology and Hepatology, Ministry of Health, Shanghai, China; ^3^ Jiangsu Key Laboratory of Molecular Medicine, Medical School of Nanjing University, Nanjing, China; ^4^ Department of Thyroid and Breast Surgery, The Affiliated Drum Tower Hospital of Nanjing University Medical School, Nanjing, China; ^5^ Department of Hepatobiliary Surgery, The Affiliated Drum Tower Hospital of Nanjing University Medical School, Nanjing, China; ^6^ Center for Public Health Research, Medical School of Nanjing University, Nanjing, China; ^7^ Department of Gerontology, The First Affiliated Hospital of Nanjing Medical University, Nanjing, China

**Keywords:** rate-limiting enzymes, lipid metabolism, gastric cancer, lymphangiogenesis, lymph node metastasis

## Abstract

Emerging evidences demonstrate that metabolic reprogramming is a hallmark of malignancies, including gastric cancer (GC). Abnormal expression of metabolic rate-limiting enzymes, as the executive medium of energy metabolism, drives the occurrence and development of cancer. However, a comprehensive model of metabolic rate-limiting enzymes associated with the development and progression of GC remains unclear. In this research, we identified a rate-limiting enzyme, sterol O-acyltransferase 1 (SOAT1), was highly expressed in cancerous tissues, which was associated with advanced tumor stage and lymph node metastasis, leading to the poor prognosis of GC. It was shown that knockdown of SOAT1 or pharmacological inhibition of SOAT1 by avasimibe could suppress GC cell proliferation, cholesterol ester synthesis, and lymphangiogenesis. However, overexpression of SOAT1 promoted these biological processes. Mechanistically, SOAT1 regulated the expression of cholesterol metabolism genes SREBP1 and SREBP2, which could induce lymphangiogenesis via increasing the expression of VEGF-C. In conclusion, our results indicated that SOAT1 promotes gastric cancer lymph node metastasis through lipid synthesis, which suggested that it may be a promising prognostic biomarker for guiding clinical management and treatment decisions.

## Introduction

Gastric cancer (GC) has the fifth highest morbidity and third highest mortality among malignancies worldwide (Sung et al., 2021). Over the last decades, improved treatments have improved the prognosis of patients with early gastric cancer. Unfortunately, GC is always diagnosed at an advanced stage with malignant proliferation and metastasis, leading to the poor prognosis of GC patients (Digklia and Wagner, 2016; Smyth et al., 2020). Therefore, it is urgent to explore novel therapeutic targets and molecular mechanisms responsible for the progression of GC.

The emerging view of cancer is that metabolic reprogramming evolves as tumors progress from precancerous lesions to locally invasive cancer to metastatic tumors (Faubert et al., 2020). Dysregulated metabolic activities can be exploited to diagnose, monitor, and treat malignancies. One of the main features of these metabolic alterations is the enhanced glycolysis and decreased mitochondrial aerobic respiration even in the presence of abundant oxygen (Warburg effect), which is a driving force of cancer cell survival, growth and aggressiveness (Vander Heiden et al., 2009; Liberti and Locasale, 2016). In addition, numerous studies have highlighted the intricate relationship between oncogenic signaling and lipid metabolism reprogramming (Snaebjornsson et al., 2020). Enhanced synthesis and uptake of lipids contribute to tumor formation and progression, dysregulation of Sterol regulatory element-binding proteins (SREBPs) plays a central role in these processes (Cheng et al., 2015). Collectively, a comprehensive understanding of the molecular mechanisms of metabolic reprogramming is essential for developing more prognostic biomarkers and therapeutic strategies.

Metabolic rate-limiting enzymes are the executive agents of energy metabolism, and abnormal changes in metabolic enzymes drive the progression of tumors. Recently, several metabolic rate-limiting enzymes in glycolysis, glutamine metabolism and fatty acid oxidation (FAO) have been identified as biomarkers and drug targets (Tong et al., 2009; Roberts and Miyamoto, 2015; Menendez and Lupu, 2017). In these processes, a critical regulatory role is played by Sterol O-acyltransferase 1 (SOAT1), one of rate-limiting enzymes of the mevalonate pathway, main function is converting excess cholesterol into cholesterol esters and stored in cytosolic lipid droplets (Cheng et al., 2018). Recent studies showed that SOAT1 is highly upregulated in malignancies and correlates inversely with patient prognosis (Geng et al., 2016; Jiang et al., 2019; Xu et al., 2020). In addition, numerous inhibitors targeting rate-limiting enzymes are in preclinical and clinical studies for different human cancers have been found to simultaneously suppress tumor growth and metastasis (Vander Heiden and DeBerardinis, 2017). However, the abnormal alterations and biological functions of rate-limiting enzymes in GC are unintelligible. Therefore, systematic identification of enzymes from metabolic rate-limiting enzyme databases may provide more potential novel anticancer treatments for GC.

In this study, we identified SOAT1 as a critical biomarker of gastric cancer, it was markedly upregulated in gastric cancer tissues, which was significantly associated with the clinicopathological characteristics and prognosis of gastric cancer patients. SOAT1 overexpression enhanced the ability of proliferation, migration and invasion of GC cells. Furthermore, SOAT1 induced SREBP1 and SREBP2 expression participated in the pro-lymphangiogenic process via promoting VEGFC expression and ultimately contributed to gastric cancer lymphangiogenesis. Importantly, pharmacological inhibition of SOAT1 by avasimibe suppressed these processes in a dose-dependent manner. Overall, our study identified the biological roles of SOAT1 in GC and uncovered that SOAT1 may be a novel biomarker and therapeutic target for GC lymph mode metastasis.

## Results

### Identification of SOAT1 as a Biomarker in GC Based on Database Analysis

**FIGURE 1:**
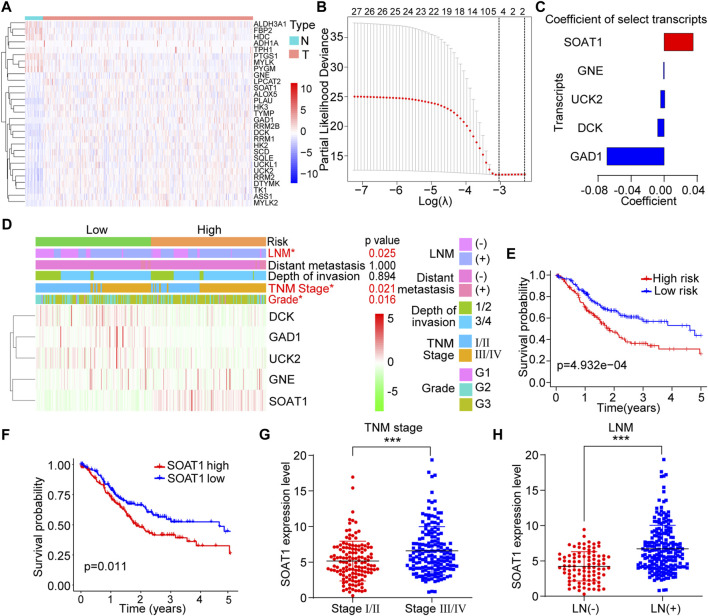
Identification of SOAT1 as a biomarker in GC from 111 rate-limiting enzymes based on TCGA database. **(A)** Heatmap of differentially expressed rate-limiting enzymes between GC tumor (*n* = 375) and normal tissues (*n* = 32) in TCGA. **(B)** The coefficients were calculated by multivariate Cox regression using the LASSO method. **(C)** Coefficients of the five selected genes. **(D)** The expression levels of the five selected genes in high- and low-risk GC patients. **(E)** Kaplan–Meier overall survival (OS) curve for GC patients assigned to the high- and low-risk groups. **(F)** Kaplan–Meier OS curve of GC patients stratified by SOAT1 expression. **(G,H)** Correlation of SOAT1 expression with TNM stage **(G)** and LNM **(H)** in GC tissues based on TCGA data.

To investigate changes in metabolic rate-limiting enzymes in GC, we systematically screened the transcriptome profiles of 111 rate-limiting enzymes between tumor and adjacent normal tissues from a database reported previously (Wang et al., 2019). All these genes were listed in the Supplementary Table S1. A total of 29 differentially expressed transcripts were identified (*p* < 0.05): 21 upregulated and 8 downregulated transcripts (Figure 1A). We then utilized Cox regression analysis with the LASSO algorithm to determine the effects of these transcripts on clinical prognosis, and five genes (SOAT1, GNE, UCK2, DCK, and GAD1) were selected according to the minimum criteria and the regression coefficients (Figure 1B). Based on the expression levels of the five genes, the following formula was derived to calculate the risk score for predicting the prognosis of each patient: Risk score = (0.0360 × expression value of SOAT1) + (−0.0006 × expression value of GNE) + (−0.0052 × expression value of UCK2) + (−0.0087 × expression value of DCK) + (−0.0700 × expression value of GAD1) (Figure 1C). By the risk score formula, the patients were divided into the low-risk (*n* = 152) and high-risk (*n* = 151) subgroups based on the mean risk score. The expression level of SOAT1 was higher in the high-risk group, while the expression levels of the other four genes were higher in the low-risk group. In addition, the high-risk group patients often had a more advanced TNM stage (*p* = 0.021), higher tumor grade (*p* = 0.016), and higher incidence of lymph node metastasis (LNM) (*p* = 0.025) (Figure 1D). Moreover, the patients in the high-risk group had shorter overall survival (OS) time than those in the low-risk group (Figure 1E). Among the five genes, high expression level of SOAT1 was significantly correlated with poor prognosis in GC patients, and the expression of the other four genes was not associated with the survival of GC patients (Figure 1F; Supplementary Figures S1A–D). Furthermore, Kaplan-Meier plotter data also confirmed that high level of SOAT1 was associated with poor survival in patients with gastric adenocarcinoma patients (Supplementary Figure S1E). Moreover, the expression level of SOAT1 was significantly associated with clinicopathological features, such as TNM stage (*p* < 0.001) and LNM (*p* < 0.001) (Figures 1G,H). However, SOAT1 expression had no statistically significant association with tumor grade, invasion depth or distant metastasis status (Supplementary Figures S1F–H). Taken together, these results revealed that the expression of SOAT1 was upregulated in GC and it might be an independent prognostic risk factor in GC patients.

### SOAT1 Is Overexpressed and Associated With Poor Prognosis in GC Patients

**FIGURE 2:**
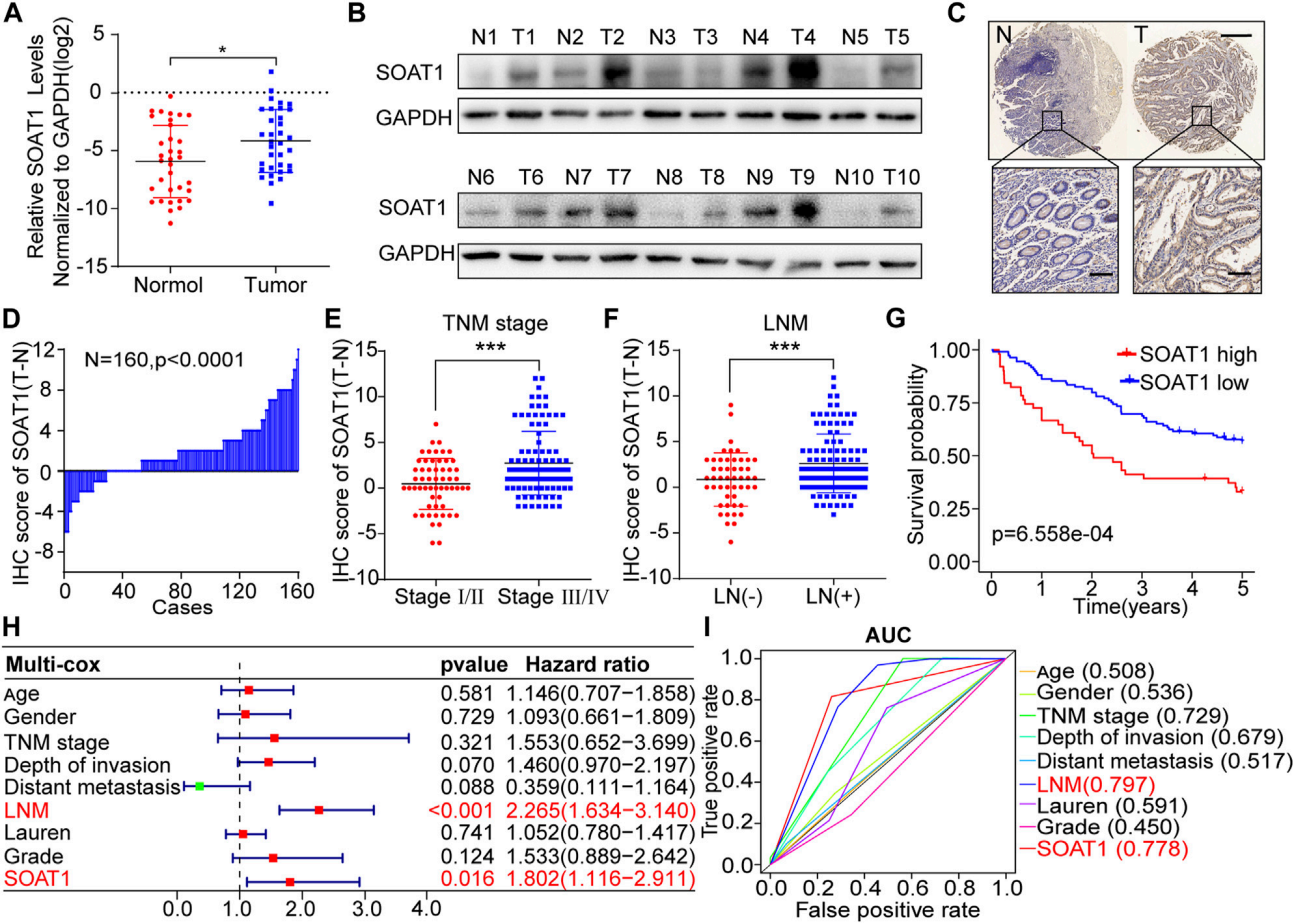
SOAT1 expression is elevated in GC and correlates with poor prognosis in GC patients. **(A)** The relative RNA expression level of SOAT1 in GC and corresponding noncancerous tissues was measured by qRT-PCR (*n* = 34). **(B)** The protein level of SOAT1 was measured by western blot assay in GC tissues and the corresponding noncancerous tissues (*n* = 10). **(C)** Representative IHC images of the TMA probed with the anti-SOAT1 antibody (scale bars = 200 and 50 μm, respectively) were shown. **(D)** The distribution of the differences in the immunoreactivity score (IRS) for SOAT1 staining was available for 160 pairs of GC tissues and the corresponding noncancerous tissues (ΔIRS = IRST-IRSN). **(E,F)** Correlation of SOAT1 expression with TNM stage **(E)** and LNM **(F)** in GC tissues based on TMA data. **(G)** Kaplan-Meier OS analysis of GC patients stratified by the relative SOAT1 expression level. **(H)** Multivariate analyses were performed with the above-mentioned GC patients, and all bars correspond to 95% confidence intervals (CIs) and hazard ratios (HRs). **(I)** Receiver operating characteristic (ROC) analysis of the risk factors in the above-mentioned GC patients.

To further confirm the expression of SOAT1 in GC, we first examined the SOAT1 expression levels in 34 GC cancerous tissues and paired adjacent noncancerous tissues. We found that the mRNA levels of SOAT1 were significantly higher in cancerous tissues (Figure 2A) and the protein levels of SOAT1 were significantly higher in 9/10 (90%) cancerous tissues than the corresponding noncancerous tissues (Figure 2B). We then utilized an independent validation cohort to further investigate SOAT1 expression and its relationship with clinical outcome using immunohistochemistry (IHC) staining in a GC tissue microarray (TMA). Similarly, these results showed that the expression of SOAT1 was significantly increased in cancerous tissues compared with the matched normal tissues (*n* = 160, *p* < 0.01; Figures 2C,D, Supplementary Figure S2B). Furthermore, the protein expression of SOAT1 in the GC cohort was significantly correlated with LNM status (*p* = 0.001), TNM stage (*p* = 0.029) and differentiation degree (*p* = 0.006); however, SOAT1 expression had no statistically significant correlation with depth of invasion (*p* = 0.195), distant metastasis (*p* = 0.328), tumor diameter (*p* = 0.697) or histological type (*p* = 0.366) (Supplementary Table S2). Moreover, SOAT1 protein expression was greatly increased in advanced-stage and LNM-positive patients (Figures 2E,F). Kaplan-Meier survival analysis showed that GC patients with high level of SOAT1 had worse OS (*p* = 6.558e-04) (Figure 2G). Simultaneously, univariate Cox regression analysis revealed that TNM stage (HR = 5.817, 95% CI: 3.114–10.836), depth of invasion (HR = 2.499, 95% CI: 1.808–3.454), LNM (HR = 2.832, 95% CI: 2.189–3.665) and SOAT1 (HR = 2.437, 95% CI: 1.567–3.791) were significantly associated with the survival in patients with GC (Supplemental Figure S2A). Furthermore, by using multivariate Cox regression analysis, we found that SOAT1 was one of the independent predictors of the prognosis of GC patients HR = 1.802 (1.116–2.911) (Figure 2H). To further evaluate the predictive ability of the SOAT1 expression level, we conducted receiver operating characteristic curve (ROC) analysis, and the area under the curve (AUC) of SOAT1 and LNM were 0.778 and 0.797, respectively (Figure 2I). Collectively, these data revealed that SOAT1 expression level was significantly increased in GC tissues and that SOAT1 might be an independent prognostic risk factor for GC.

### SOAT1 Promotes GC Cells Proliferation, Migration and Invasion

**FIGURE 3:**
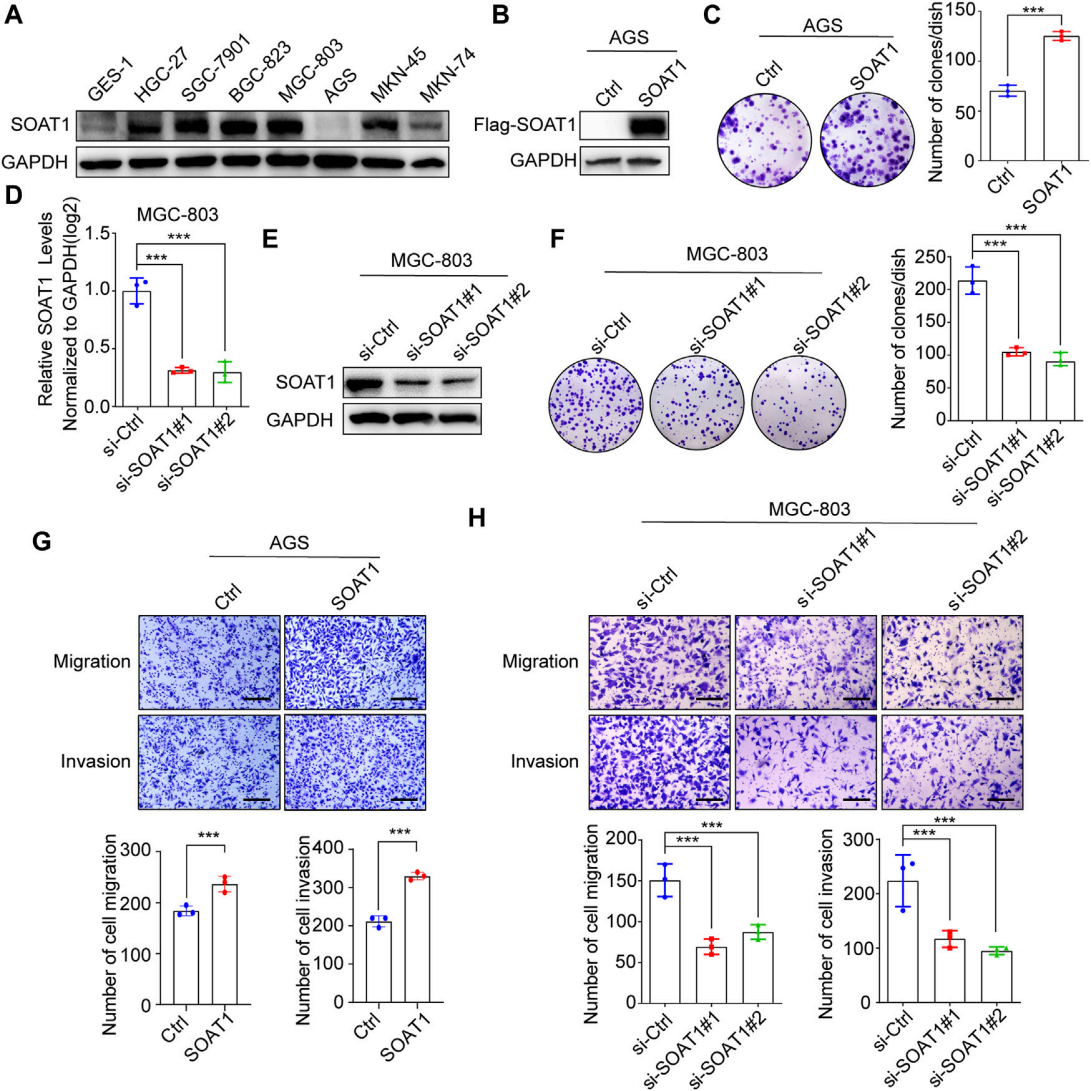
SOAT1 promotes GC cell proliferation, migration and invasion. **(A)** The protein level of SOAT1 in GES-1 cells and GC cell lines were analyzed by western blot. **(B)** The overexpression efficiencies were verified at the protein level in AGS by western blot. **(C)** Representative images of colony formation assay in AGS after overexpression of SOAT1 (left panel), the right panel shows the quantitative results. **(D,E)** The knockdown efficiency was verified at the mRNA and protein levels by qRT-PCR **(D)** and western blot assay **(E)**, respectively. **(F)** Representative images of colony formation assay in MGC-803 after knockdown of SOAT1 (left panel), and the quantitative data were shown (right panel). **(G)** Transwell assays in SOAT1 overexpression AGS cells. Representative images of migrated and invaded GC cells in each group were shown in the upper panel, and quantitative results were shown in the lower panel. **(H)** Transwell assay in MGC-803 cells after knockdown of SOAT1. Representative images of migrated and invaded GC cells in each group were shown in the left panel, and the quantitative results were shown in the right panel. Scale bars: 100 µm.

To elucidate the function of SOAT1 in GC cells, we first investigated SOAT1 expression in GC cell lines and normal gastric mucosal cells (GES-1) by western blot, and it was shown that its expression was higher in most of GC cells than GES-1 cells (Figure 3A). Subsequently, we established stable SOAT1-overexpressing GC cells in AGS cells with relative low expression of SOAT1 (AGS-SOAT1) (Figure 3B). As shown in Figure 3C, overexpression of SOAT1 significantly increased the colony formation efficiency of AGS cells. We also knocked down SOAT1 by two specific siRNAs in MGC-803 and BGC-823 cells and confirmed that both the mRNA and protein levels of SOAT1 were markedly reduced after transfection for 72 h (Figures 3D,E; Supplementary Figures S3A–B). As expected, knockdown of SOAT1 obviously decreased the colony formation efficiency (Figure 3F; Supplementary Figure S3C). All these results confirmed that SOAT1 promoted the proliferative ability of GC cells.

In addition, transwell analysis was performed to determine the role of SOAT1 in GC cell migration and invasion. The results showed that over expression of SOAT1 promoted the migration and invasion of AGS cells (Figure 3G). Conversely, knockdown of SOAT1 in MGC-803 and BGC-823 cells exerted the opposite effects (Figure 3H; Supplementary Figure S3D). Collectively, these data indicate the critical role of SOAT1 in promoting GC cell proliferation, migration and invasion.

### Inhibitory Effects of Avasimibe on the Proliferation and Metastasis of GC Cells

**FIGURE 4:**
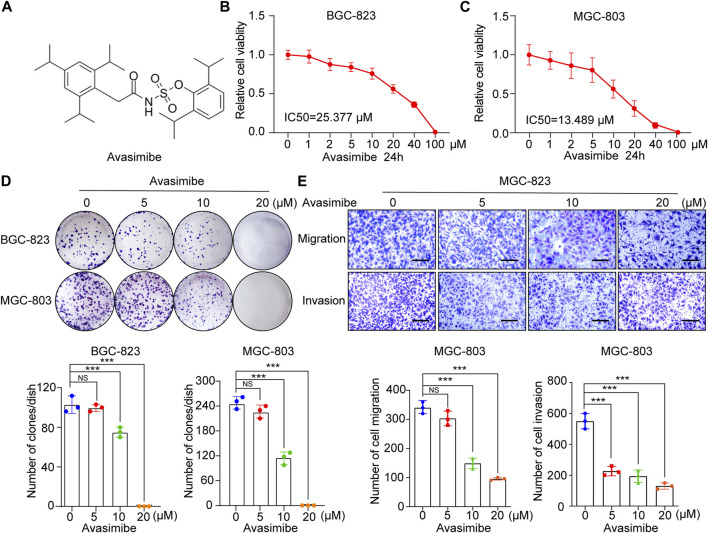
Avasimibe inhibits GC cell growth and metastasis. **(A)** Molecular structure of avasimibe. **(B,C)** Relative cell viability was measured by CCK-8 assay after avasimibe treatment for 24 h at multiply concentrations (0, 1, 2, 5, 10, 20, 40, and 100 μM). **(D)** Representative images of colony formation assay in BGC-823 and MGC-803 cells after avasimibe treatment (upper panel); the quantitative results were shown (lower panel). **(E)** Transwell assay in MGC-803 cells after avasimibe treatment. Representative images of migrated and invaded GC cells in each group were shown in the left panel, and the quantitative results were shown in the right panel. Scale bars: 100 µm.

Avasimibe, a potent small molecule inhibitor of SOAT1 (Figure 4A), has been proven to exert anticancer effects against many tumors, including glioblastoma (GBM), hepatocellular carcinoma (HCC) and prostate cancer (PCa) (Tardif et al., 2004; Bemlih et al., 2010; Jiang et al., 2019; Liu et al., 2021). Herein, we investigated whether avasimibe has an inhibitory effect on GC cells. As shown by the cell viability curve, avasimibe inhibited the proliferation of GC cell lines in a dose-dependent manner. The IC50 values of avasimibe in MGC-803 and BGC-823 cells at 24 h were 13.489 and 25.377 μM, respectively. In addition, the respective IC50 values at 48 h were 8.811 and 14.208 μM (Figures 4B,C; Supplementary Figures S4A,B). Consistent with the CCK-8 assay results, avasimibe also decreased the colony-forming capacity of GC cells in a dose-dependent manner (Figure 4D).

Next, we explored the effects of avasimibe treatment on the cell metastasis ability in GC cell lines. The wound healing assay showed that avasimibe significantly reduced the migration capability of BGC-823 cells (Supplementary Figure S4C). In addition, we performed a transwell assay, which showed significant dose-dependent decreases in the numbers of migrated and invaded MGC-803 and BGC-823 cells after treatment with avasimibe (Figure 4E; Supplementary Figure S4D). Collectively, these results indicated the critical inhibitory effect of avasimibe on GC cell proliferation and metastasis.

### SOAT1 Accelerates Lipid Metabolism in GC Cells

**FIGURE 5:**
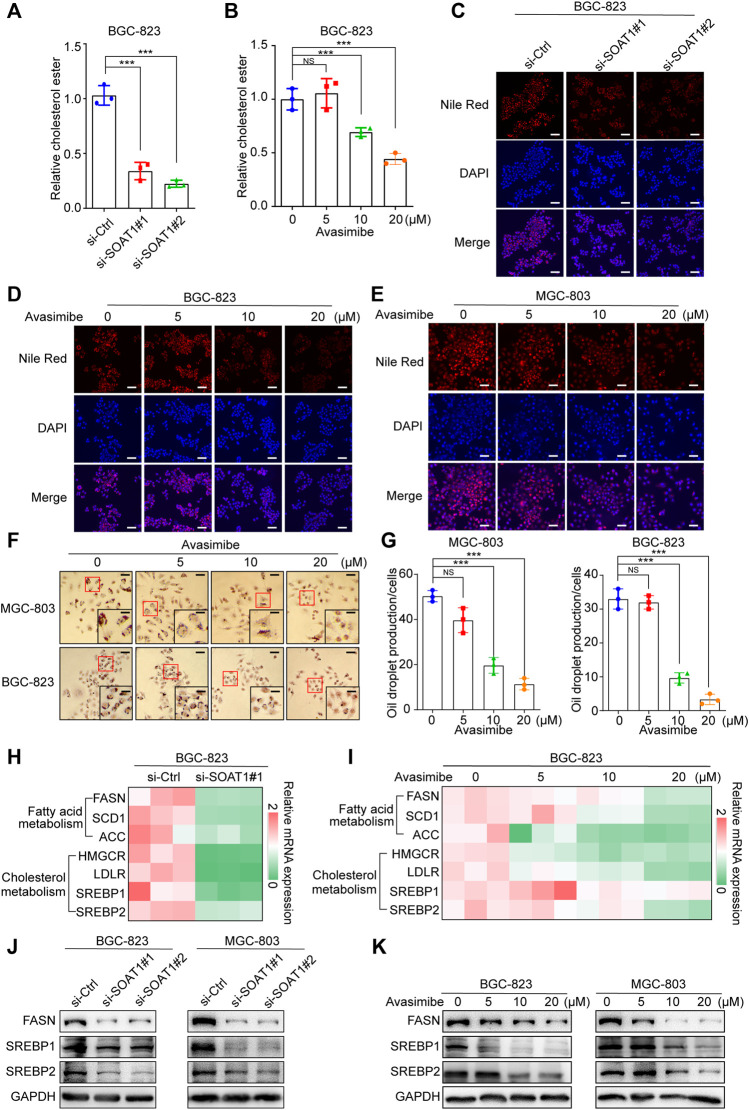
Inhibition of SOAT1 decreases the level of lipid synthesis in GC cells. **(A,B)** Cholesterol ester assays for BGC-823 cells after knockdown of SOAT1 **(A)** or avasimibe treatment **(B)**. **(C)** Representative images of Nile red stanning assay in BGC-823 cells after knockdown of SOAT1. Scale bars: 100 µm. **(D, E)** Representative images of Nile red stanning assay in BGC-823 **(D)** and MGC-803 **(E)** cells after avasimibe treatment. Scale bars: 100 µm. **(F)** Representative images of Oil red O staining in BGC-823 and MGC-803 cells after avasimibe treatment. **(G)** Quantitative data for lipid synthesis were shown. Scale bars: 100 and 50 μm, respectively. **(H,I)** Heatmap generated from the qRT-PCR results showed the gene expression levels of cholesterol metabolism and fatty acid biosynthesis-related genes in BGC-823 cells after knockdown of SOAT1 **(H)** or avasimibe treatment **(I)**. **(J,K)** FASN, SREBP1, and SREBP2 protein expression were measured by western blot assay after knockdown of SOAT1 **(J)** or avasimibe treatment **(K)**.

It has been reported that SOAT1 can convert excess free cholesterol into cholesteryl esters for storage in lipid droplets (Xu et al., 2020). We first detected the level of cholesterol in GC cells, and the results showed that esterified cholesterol was significantly reduced in SOAT1 knocked-down GC cells or avasimibe treatment (Figures 5A,B). In addition, we evaluated whether the inhibition of SOAT1 affected the formation of lipid droplets by Nile red and oil red O staining. The results showed that knocking down SOAT1 could significantly reduce the number of lipid droplets in GC cells (Figure 5C, Supplementary Figures S5A,B), and avasimibe treatment had a similar effect (Figures 5D–G).

To further confirm that SOAT1 regulates lipid biosynthesis and catabolism, we examined the expression levels of a panel of lipid metabolism-related genes in SOAT1-knockdown and avasimibe-treated GC cells. Intriguingly, both SOAT1 knockdown and avasimibe treatment reduced the expression of cholesterol metabolism genes (HMGCR, SREBP1, and SREBP2) and fatty acid biosynthesis genes (FASN, ACC, and SCD1) (Figures 5H,I; Supplementary Figures S5C,D). In addition, the expression of SREBP1, SREBP2, and FASN were strikingly reduced at the protein level (Figures 5J–K). Taken together, these results suggested that dysregulated SOAT1 accelerates the esterification of cholesterol and the synthesis of lipids in GC cells.

### SOAT1 Is Related to Lymph Node Metastasis in GC Patients and Promotes Lymphangiogenesis

**FIGURE 6:**
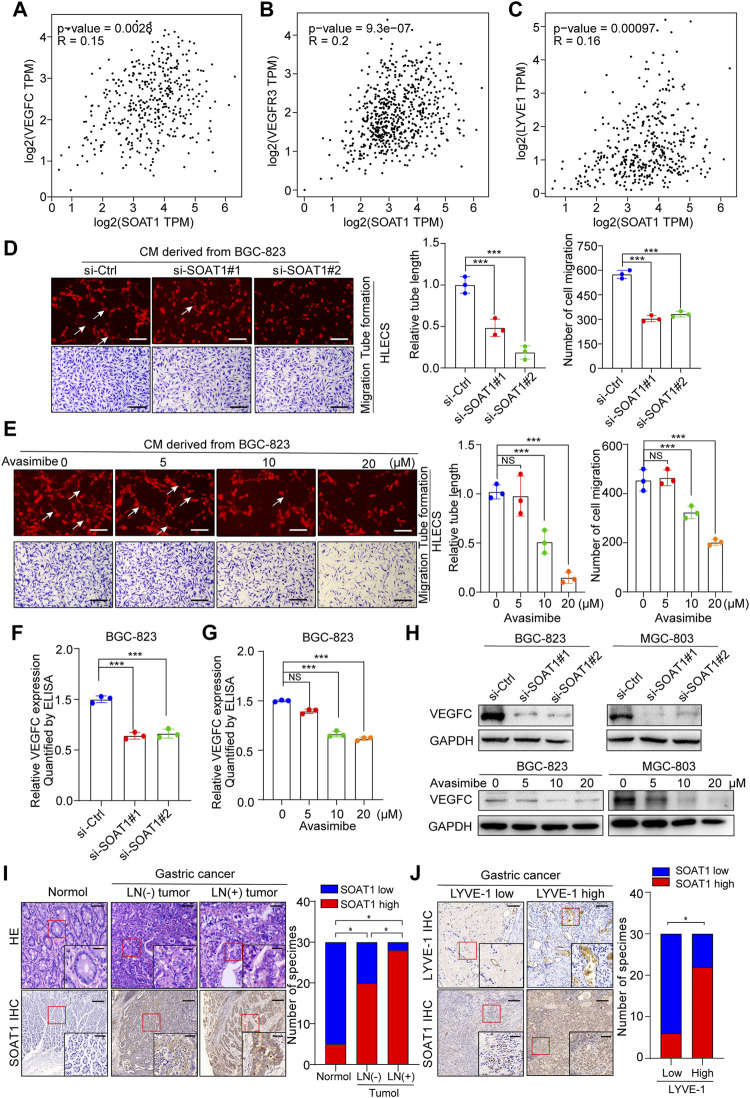
SOAT1 promotes lymphangiogenesis and lymph node metastasis in GC. **(A–C)** The correlation of SOAT1 expression with VEGFC, VEGFR3, and LYVE-1 were analyzed via online bioinformatics tool (http://gepia.cancer-pku.cn/). **(D,E)** Representative images (left panel) and quantitative analysis (right panel) of tube formation and transwell migration assays of HLECs cultured with conditioned medium collected from SOAT1-knockdown **(D)** or avasimibe-treated **(E)** BGC-823 cells. Scale bars: 100 µm **(F,G)** ELISA assay of VEGFC expression in conditioned medium collected from the SOAT1 knockdown or avasimibe treated BGC-823 cells. **(H)** VEGFC expression at the protein level in BGC-823 and MGC-803 cells were measured by western blot assay after knockdown of SOAT1 or avasimibe treatment. **(I)** Representative images (left panel) and quantitative analysis (right panel) of SOAT1 IHC staining in paraffin-embedded paired normal tissues, sections of tumors with or without LNM from patients with GC. Scale bars: 200 µm and 50 μm, respectively. **(J)** Representative images (left panel) and quantitative analysis (right panel) of SOAT1 IHC staining in high or low levels of LYVE-1-positive microvessels in the GC tissues. Scale bars: 200 µm and 50 μm, respectively.

It has been demonstrated that lipids are required as energy sources and cellular signaling molecules, which are crucial for cancer lymphangiogenesis and lymph node metastasis (Ma Y. et al., 2018; Lee et al., 2019). In addition, the bioinformatic analysis using TCGA and our TMA data suggested that the expression level of SOAT1 was significantly higher in LNM-positive GC tissues (Figures 1H, 2F). Thus, we speculated whether SOAT1 could promote lymph node metastasis of GC cells. VEGFC, VEGFR3, and LYVE-1, which play key role in lymph node metastasis of multiple malignancies, therefore we used online bioinformatics tools (http://gepia.cancer-pku.cn/) to study the relationship between SOAT1 and VEGFC, VEGFR3, and LYVE-1 expression. The results showed that the expression level of SOAT1 was significantly positively correlated with VEGFC, VEGFR3, and LYVE-1 (Figures 6A–C). Next, we further examined whether SOAT1 has an effect on tumor-induced lymphangiogenesis. As shown in Figures 6D,E and Supplementary Figures S6A,B, HLECs treated with conditioned medium (CM) derived from SOAT1-knockdown or avasimibe treatment GC cells significantly reduced the lymphatic capillary formation and the migratory capability of HLECs compared with CM derived from the corresponding vector cells. In addition, compared with CM from the control group, CM from SOAT1-overexpressing GC cells significantly promoted these biological processes (Supplementary Figure S6C). As expected, qRT-PCR data showed that the mRNA levels of VEGFC and VEGFD were significantly decreased in both SOAT1-knockdown and avasimibe-treated GC cells (Supplementary Figures S6D–G). Furthermore, we evaluated the secretion level of VEGFC using ELISA, and found that the VEGFC protein levels were significantly decreased in conditioned medium collected from GC cells which knockdown of SOAT1 or treated with avasimibe compared to conditioned medium from the corresponding control cells (Figures 6F,G; Supplementary Figures S6H,I). In addition, the expression of VEGFC were also decreased at the protein levels (Figure 6H). Intriguingly, the IHC staining results showed that the expression of SOAT1 was scarcely detected in adjacent noncancerous tissues, slightly increased in LNM-negative GC tissues and strongly upregulated in LNM-positive GC tissues, and positively correlated with the density of microlymphatic vessels, as indicated by the number of LYVE-1-positive microvessels (Figures 6I,J). Taken together, these findings indicated that inhibition of SOAT1 suppresses lymphangiogenesis and that avasimibe has the potential therapeutic effect for GC patients with lymph node metastasis.

### SOAT1 Accelerates the Lymphangiogenesis by Activating SREBP1 and SREBP2

**FIGURE 7:**
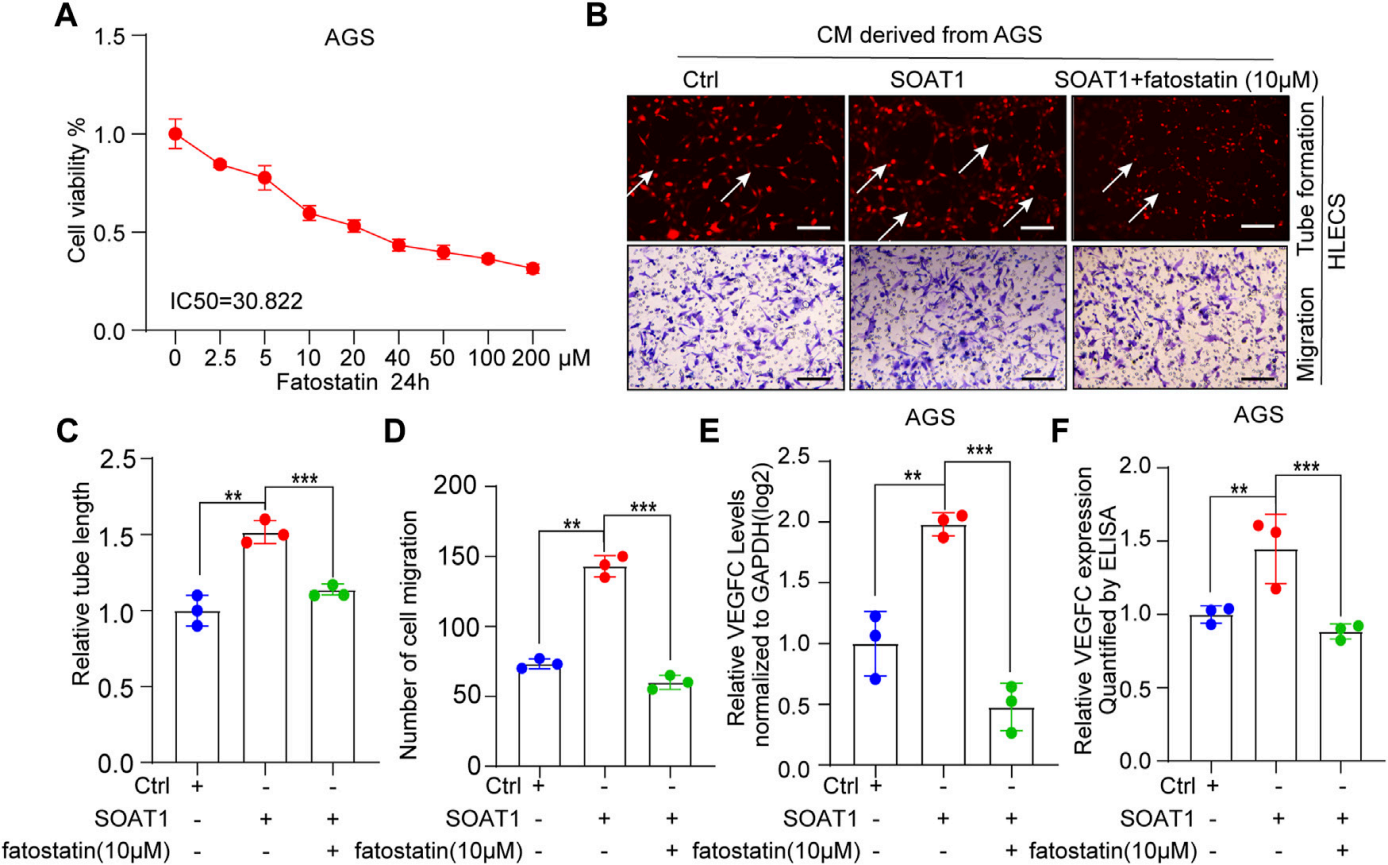
SOAT1 accelerates GC lymphangiogenesis by upregulating the activity of SREBP1 and SREBP2. **(A)** Relative cell viability was measured by CCK-8 assay after fatostatin treatment for 24 h at multiply concentrations (0, 2.5, 5, 10, 20, 40, 100, and 200 µM). **(B–D)** Representative images **(A)** and quantification results **(C,D)** of the tube formation and transwell migration assays of HLECs cultured with conditioned medium collected from SOAT1-overexpressing AGS cells treated with fatostatin or the corresponding controls. **(E)** The expression level of VEGFC was measured by qRT-PCR in SOAT1-overexpressing AGS cells treated with fatostatin or their corresponding controls. **(F)** ELISA assay of VEGFC expression in conditioned medium collected from the SOAT1-overexpressing AGS cells treated with fatostatin or their corresponding controls.

SREBP1 and SREBP2 are both important signaling molecules that relate to tumor lymph node metastasis (Heo et al., 2020; Li et al., 2020). To investigate whether SREBP1 and SREBP2 involved in SOAT1-mediated lymphangiogenesis and VEGFC production, we pharmacologically inhibited SREBP1 and SREBP2 by fatostatin, which displays antitumor activity in cancers by downregulating SREBP- mediated metabolic pathways (Li et al., 2014). As shown by the cell viability curve, fatostatin inhibited the proliferation of AGS cells in a dose-dependent manner and the IC50 value is 30.822 µM (Figure 7A). Intriguingly, the results showed that fatostatin could significantly reversed the promoting effects of SOAT1 overexpression on the migration and the lymphatic tube formation of GC cells (Figures 7B–D). In addition, ELISA and qRT-PCR results suggested that the expression of VEGFC were recovered after treated with fatostatin (Figures 7E,F). Collectively, these results indicating that blocking SREBP1 and SREBP2 pathway inhibits SOAT1-mediated lymphangiogenesis in gastric cancer.

## Discussion

Recently, accumulating investigations have confirmed that metabolic reprogramming plays a significant role in malignant processes in various cancers (Li et al., 2019). Rate-limiting enzymes play a vital role in malignant processes, and cancer cells tend to have a robust metabolism and increase their energy consumption by changing these rate-limiting enzymes (Faubert et al., 2020; Xu et al., 2020). For instance, Hexokinase 2 (HK-2), the rate-limiting enzyme in glycolysis, decreases mTORC1 activity and regulates autophagy through direct phosphorylation of ULK1 (Roberts et al., 2014; Roberts and Miyamoto, 2015). Renal glutaminase (GLS1) provides the antioxidants glutathione (GSH) and nicotinamide adenine dinucleotide phosphate (NADPH) for tumor cell metabolism, and promotes tumor cell growth by reducing reactive oxygen species (ROS) (Tong et al., 2009). High HMGCR activation decreases the growth-inhibitory effect of atorvastatin on TGF-β-treated epithelial cancer cells (Warita et al., 2021). We systematically screened the expression of 111 rate-limiting enzymes in TCGA database, and identified SOAT1 expression level is significantly increased in cancer tissues and closely associated with the poor outcome of GC patients. Subsequently, our TMA data also confirmed that SOAT1 expression is associated with the clinicopathological characteristic and prognosis in patients. More importantly, SOAT1 expression level have a powerful predictive ability of clinical risk scores. Thus, SOAT1 could be used as an effective potential predictive biomarker and therapeutic target for GC.

SOAT1, localized in the endoplasmic reticulum, catalyzes the formation of cholesterol esters (Chang et al., 2006; Chang et al., 2009). SOAT1 is frequently upregulated in multiple cancers, including GBM, HCC and PCa (Geng et al., 2016; Gu et al., 2020; Liu et al., 2021). Previous research demonstrated that SOAT1 promotes lipid metabolism and tumor growth (Jiang et al., 2019; Gu et al., 2020). In addition, Oni et al. (2020) reported that SOAT1 promotes organoid growth and tumor metastasis in pancreatic carcinoma mouse model by activating the mevalonate pathway and disrupting the negative feedback of cholesterol. However, the biological function of SOAT1 and its regulatory mechanisms in GC remain elusive. Our experiment data demonstrated that SOAT1 overexpression elevated GC cell proliferation, migration and invasion ability, highlighting the role of SOAT1 in GC progression. We also showed that inhibition of SOAT1 decreases the synthesis of cholesterol eater and the formation of lipid droplets. Mechanically, we also confirmed that inhibition of SOAT1 downregulating these genes related to numerous aspects of cholesterol metabolism and fatty acid biosynthesis.

Notably, numerous studies have demonstrated that cancer cells undergo metabolic changes during the progression of lymph node metastasis, dysregulated lipid metabolism plays an indispensable role in this process. The metabolism of cholesterol, bile acid and fatty acid are critical in the proliferation and differentiation of lymphatic endothelial cells (Wong et al., 2017). In addition, lymph node metastasis requires that tumor cells undergo a metabolic transition toward fatty acid oxidation (Wong et al., 2017; Lee et al., 2019). SOAT1 catalyzes the conversion of excess cholesterol into cholesterol esters for storage in lipid droplets, and high expression of SOAT1 in tumors may disrupt the balance between free cholesterol and cholesterol esters (Oelkers et al., 1998; Li et al., 2006). Our statistical analysis revealed that the expression level of SOAT1 was higher in LN-metastatic tumors and was positively correlated with the expression level of VEGFC, VEGFR3 and LYVE-1, which are lymphangiogenic growth factors (Ji et al., 2014; Stacker et al., 2014; Ma C. et al., 2018). We also found that inhibition of SOAT1 effectively reduced tumor-associated lymphangiogenesis and the migratory ability of HLECs. Additionally, the expression of VEGFC was dramatically decreased in GC cells after knockdown of SOAT1. Intriguingly, the promoting effect of SOAT1 on lymphangiogenesis was restored by fatostatin. Taken together, these data demonstrate that SOAT1 inhibition leads to suppression of lipid synthesis and GC lymph node metastasis by SREBP1 and SREBP2 pathway.

Rate-limiting enzymes are considered as rational targets for antitumor drug development, and inhibitors of these enzymes have applicated in the clinical trial as promising methods for malignancies, such as statins, perhexiline and trimetazidine (Tong et al., 2009; Ma Y. et al., 2018). Recently, several SOAT1 inhibitors have been discovered, including K-604, nevanimibe, and avasimibe. K-604 has been confirmed an inhibitory effect in glioblastoma cells by downregulated the activation of Akt and extracellular signal-regulated kinase (Ohmoto et al., 2015). Nevanimibe shows the significant suppresses effect in metastatic adrenocortical carcinoma (ACC) (Smith et al., 2020). Among these agents, avasimibe is a notable anticancer drug that significantly reduces cholesteryl ester storage by inhibits vesicular transport, integrin and TGF-β pathways (Jiang et al., 2019). In the current study, we first found that avasimibe notably suppressed the proliferation, migration and invasion of GC cells in a dose-dependent manner. Subsequently, avasimibe induce a decrease in cholesterol ester synthesis and lipid droplet formation in GC cells. Importantly, our data indicated that HLECs tube formation and migration ability were significantly inhibited by avasimibe. Our further investigation showed that avasimibe significantly decreased the expression and secretion of VEGFC in gastric cancer cells. These results provide that avasimibe may serve as a potential chemical inhibitor for the treatment of lymph node metastasis of gastric cancer.

In summary, our results reveal for the first time the role for SOAT1 as a biomarker for GC development and lymph node metastasis, and the antitumor effect of avasimibe on GC cells. These findings suggest that SOAT1 may be a potential predictor and therapeutic approach for the development of gastric cancer.

## Materials and Methods

### Cell Culture

Human GC cell lines AGS cells were purchased from the American Type Culture Collection (ATCC). HLECs, HEK-293T, BGC-823, HGC-27, MGC-803, SGC-7901, MKN-74, and MKN-45 were obtained from the Type Culture Collection of the Chinese Academy of Sciences (Shanghai, China). HEK-293T cells were cultured in DMEM (Biological Industries, Cromwell, CT, United States), AGS cells were cultured in F12K medium (Biological Industries, Cromwell, CT, United States), and the other cells were cultured in RPMI-1640 medium (Biological Industries, Cromwell, CT, United States). All cells were cultured with 10% fetal bovine serum (FBS; Biological Industries, Cromwell, CT, United States), 100 U/ml penicillin (Invitrogen), 100 μg/ml streptomycin (Invitrogen) and incubated in 5% CO_2_ at 37°C.

### Patients and Specimens

A total of 34 pathologically confirmed GC tissues and the corresponding adjacent noncancerous fresh frozen tissues were collected from patients treated with radical gastrectomy at the Nanjing Drum Tower Hospital, the Affiliated Hospital of Nanjing University Medical School (Nanjing, Jiangsu, China). All patients provided written informed consent, and all these tissues were obtained for further qRT-PCR, western blot and immunohistochemistry assay.

### Immunohistochemistry

A total of 160 pathologically confirmed GC patient tissues from Nanjing Drum Tower Hospital were obtained for TMA construction, and the expression of SOAT1 was detected by IHC staining. The chips were stained and scanned by Servicebio (Wuhan, Hubei, China) according to a standard protocol. Stanning of SOAT1 was scored by the two pathologists blind to the clinical data by applying a semi-quantitative immunoreactivity score (IRS), IRS of 0–6 and IRS of 8–12 were classified as low and high expression of SOAT1, respectively.

### SiRNAs and Plasmids

SOAT1 si-RNAs were designed and synthesized by RiboBio (Guangzhou, China), and the sequence was showed as follows: si-SOAT1#1: 5′-TAATGGTCGAATTGACATAA-3′; si-SOAT1#2: 5′-TTG​AAC​TCA​AGT​ACC​AGC​CTT​C-3′. The vector expressing SOAT1 (pcDNA3.1-SOAT1) as well as the blank pcDNA3.1-vector were purchased from Hanbio Bio-technology Company (Shanghai, China). SiRNA and plasmid were transfected with DharmaFECT4 (GE Healthcare) and Lipofectamine 3000 (Invitrogen, Carlsbad, CA, United States). Virus-containing supernatant was collected 48 h after transfection and was added to cell when confluence reached 70%, 48 h later, transduced cells were selected with 1 μg/ml puromycin.

### RNA Extraction and qRT-PCR Assay

Total RNAs were extracted from human GC tissues and associated non-cancerous tissues using TRIzol reagent (Invitrogen, Carlsbad, CA, United States) according to the manufacturer’s instructions. The reverse transcription reaction (RT) was performed with Reverse Transcription kit (Vazyme, Nanjing, China). The RT-PCR reactions were performed with a SYBR Green PCR Kit (Vazyme, Nanjing, China), measured in triplicate and performed on an Applied Biosystems 7900HT sequence detection system (Applied Biosystems). GAPDH was used as an internal control for mRNA. The relative expression levels of the target genes were calculated using the comparative 2−ΔΔCt method. All primers used in this study were listed in Supplementary Table S3. All results were obtained from three independent experiments performed in duplicate.

### Western Blot Assay

The Western blot protocol was performed as previously described(Wang et al., 2020). The antibodies used were listed as follows: anti-SOAT1 (Immunoway, YN1370); anti-SOAT1 (Abcam, ab39327); anti-VEGFC (Proteintech, 14517-1-AP); anti-FASN (Proteintech, 10624-2-AP); anti-SREBP1 (Proteintech, 66875-1-AP); anti-SREBP2 (Proteintech, 28212-1-AP); and anti-GAPDH (Proteintech, 60004-1-Ig). Phenylmethylsulfonyl fluoride was purchased from Selleck (Houston, TX, United States).

### Proliferation Assay

The antiproliferative effect of avasimibe and fatostatin (MedChemExpress, Shanghai, China) was evaluated with a Cell Counting Kit-8 kit (CCK-8, Vazyme, Nanjing, China). One day before avasimibe treatment, GC cells were seeded in 96-well plates (5,000 cells/well). The cells were treated with avasimibe for 24 h or 48 h. After the indicated time, 10 µL per well of CCK-8 solution was added and incubated at 37°C for 1 h. Absorbance was recorded at 450 nm, and five independent assays were carried out.

For the plate colony formation assay, GC cells were seeded in 6-well plates or 12-well plates (1,000 cells/well in 6-well plates and 500 cells/well in 12-well plates) and incubated for 10–14 days. The medium with or without avasimibe was changed every other day. Then, the cells were fixed with 4% paraformaldehyde for 15 min and stained with crystal violet for 1 h. Images were acquired with a digital camera, and three independent assays were carried out.

### Oil Red O Staining and Nile Red Staining

GC cells were seeded in 12-well plates, following by 0.2 µM oil acid (OA) (Sigma, United States) for 24 h. Then the cells were fixed with 4% paraformaldehyde solution. For Oil red O staining, cells were incubated with 60% isopropanol for 15 min before stanning by Oil red O working solution. The Oil red O working solution was prepared by diluting the Oil red O stock solution with distilled water at a ratio of 3:2, followed by filtration. For Nile red staining, cells were sequentially stained with 0.05 μg/ml Nile red (Sigma, United States), washed with PBS twice and then stained with DAPI (Beyotime, Shanghai, China). Images of the cells were acquired by fluorescence microscopy. All the operations were performed in a dark environment.

### HLECs Tube Formation and Transwell Assays

HLECs were seeded in 96-well plates (2 × 10^4^/well, precoated with 50 µL of Matrigel, Corning Life Sciences, Bedford, MA, United States), containing medium obtained from tumor cells and cultured for 12 h. Images of lymphatic tubes were acquired using a fluorescence microscope and quantified by measuring the number and area of the completed tubule structures.

Cell migration and invasion abilities were evaluated by transwell chambers (Corning Life Sciences, Bedford, MA, United States). Briefly, a total of 5 × 10^4^ GC cells suspended in media without FBS were seeded in the upper chambers coated with or without 50 μL of Matrigel (BD Biosciences). Then, 600 μL of culture medium containing 10% FBS was added to the lower chambers. After incubation at 37°C for 12 h, the cells in the lower chambers were fixed with 4% paraformaldehyde for 30 min, stained with crystal violet (Beyotime, Shanghai, China) for 30 min. Finally, three random fields were microscopically examined, and the number of cells was determined by photoshop software.

### Wound Healing Assay

GC cells were seeded into six-well plates and grown to 90% confluence. The cell layer was scrapping with a 10-μL sterile pipette tip and washed with three times with PBS to remove the floating and detached cells, then cultured in 1% FBS medium and images were acquired under a microscope at multiple time points (0, 6, 12, and 24 h).

### Enzyme-Linked Immunosorbent Assay

The cell culture supernatant was collected, secreted VEGFC was quantified using the Human VEGFC Quantikine ELISA Kit (Cat. No. E-EL-H1600c, elabacience) according to the manufacturer’s instructions. Briefly, GC cells seeded in 6-well culture plates were cultured in the growth medium until 90% confluence. The cells were washed three times with phosphate-buffered saline (PBS) and cultured in serum-free medium for 24 h. The cell culture supernatant or VEGFC standards were added to the 96-well plates coated with polyclonal antibody specific for human VEGFC in triplicate and incubated for 1.5 h at 37°C. Then, the Biotinylated detection Ab working fluid was added to each well and incubated for 1 h at 37°C. Afterwards, the well was washed five times, HRP conjugate working solution was added and incubated for 30 min at 37°C. Then, substrate solution was added to each well, and incubated for 30 min at 37°C in the dark. Then, the stop solution was added to each well. Absorbance was determined at 450 nm. All assays were performed in triplicate.

### Cholesterol Assay

Cells were collected by centrifugation for 10 min (4°C, 1,000×*g*) and resuspended in 200 μL extracting solution, then ultrasonic crushing in an ice bath was performed for 30 s. Supernatants were combined and centrifuged for 20 min at 10,000 × *g* (4°C) and placed on ice for measurement. Total cholesterol and unesterified cholesterol were quantitated using the manufacturer’s protocol of a Total Cholesterol and Cholesterol ester Fluorescence Determination Kit (Cat. No. E-BC-F032, elabacience). Amount of cholesterol ester were determined by subtracting the amount of unesterified cholesterol from total cholesterol.

### Statistical Analysis

A total of 111 human rate-limiting metabolic enzymes were obtained from the rate-limiting enzyme database according to a previous study (Wang et al., 2019). The RNA expression data and clinical information of GC patients were obtained from TCGA (https://tcga-data.nci.nih.gov/tcga/). Differentially expressed genes were screened with the R package “limma.” The expression level of prognostic associated rate-limiting metabolic enzymes between cancerous and normal samples was displayed via package “heatmap” and “ggplot,” respectively. Univariate Cox regression analysis was performed to identify prognostic associated rate-limiting metabolic enzymes. Package “glmnet” was used to perform LASSO Cox regression model to select optimal weighting coefficients via penalized maximum likelihood and build a prognostic signature. The formula of the risk score for the prediction of GC patients’ prognosis was as follows: risk score = the sum of the multivariate Cox regression coefficient ratio of each mRNA multiplied by the expression level of each mRNA. For survival analysis, overall survival was defined as the time from first treatment to death for any cause, Kaplan-Meier method and log-rank test were used to detect potential prognostic factors. For clarify relationship of SOAT1, clinicopathological characteristics, and prognosis, univariate Cox regression analysis was performed to find out the independent factors correlated with OS. AUC was employed to demonstrate the sensitivity and specificity of different variables by risk estimation. The “pROC” package was used to perform ROC curve and analyze AUC. All statistical tests were two sided and *p* < 0.05 were significant and each experiment was carried out in at least triplicate.

## Data Availability

The original contributions presented in the study are included in the article/Supplementary Material, further inquiries can be directed to the corresponding authors.

## Glossary

GC: Gastric cancer
SOAT1: Sterol O-acyltransferase 1
LNM: Lymph node metastasis
NADPH: Nicotinamide adenine dinucleotide phosphate
GSH: Glutathione
FAO: Fatty acid oxidation
FASN: Fatty acid synthase
KGA: Kidney-type glutaminase
EMT: Epithelial–mesenchymal transition
HLECs: Human lymphatic endothelial cells
TCGA: The cancer genome atlas
GNE: Glucosamine
UCK2: Uridine-cytidine kinase 2
DCK: Deoxycytidine kinase
GAD1: Glutamate decarboxylase 1
OS: Overall survival
IHC: Immunohistochemistry
TMA: Tissue microarray
HR: Hazard ratio
CI: Confidence interval
ROC: Receiver operating characteristic curve
AUC: Area under the curve
GBM: Glioblastoma
HCC: Hepatocellular carcinoma
PCa: Prostate cancer
IC50: Half-inhibitory concentration
OA: Oil acid
HMGCR: 3-Hydroxy-3-Methylglutaryl-CoA Reductase
SREBP1: Sterol regulatory element binding transcription factor 1
SREBP2: Sterol regulatory element binding transcription factor 2
ACC: Acetyl-CoA carboxylase 1
SCD1: Stearoyl-CoA desaturase 2
VEGFC: Vascular endothelial growth factor C
VEGFD: Vascular endothelial growth factor D
VEGFR3: Vascular endothelial growth factor receptor 3
LYVE-1: Lymphatic vessel endothelial hyaluronan receptor 1
CM: Conditioned medium
qRT-PCR: Real-time quantitative reverse transcription
HK-2: Hexokinase 2
mTORC1: mammalian target of rapamycin
Ulk1: Unc-51 like autophagy activating kinase 1
GLS1: Glutaminase 1
ROS: Reactive oxygen species
LECs: Lymphatic endothelial cells
TGF-β: Transforming growth factor beta 1.
