# Supplementary material for: SOAT1 Promotes Gastric Cancer Lymph Node Metastasis Through Lipid Synthesis
Source: Front Pharmacol. 2021 Nov 1;12:769647. doi: 10.3389/fphar.2021.769647 (PMC8591064; doi:10.3389/fphar.2021.769647)
Supplement: Supplementary file 1 [file DataSheet1.docx]

Supplementary Material

# Supplementary Figures


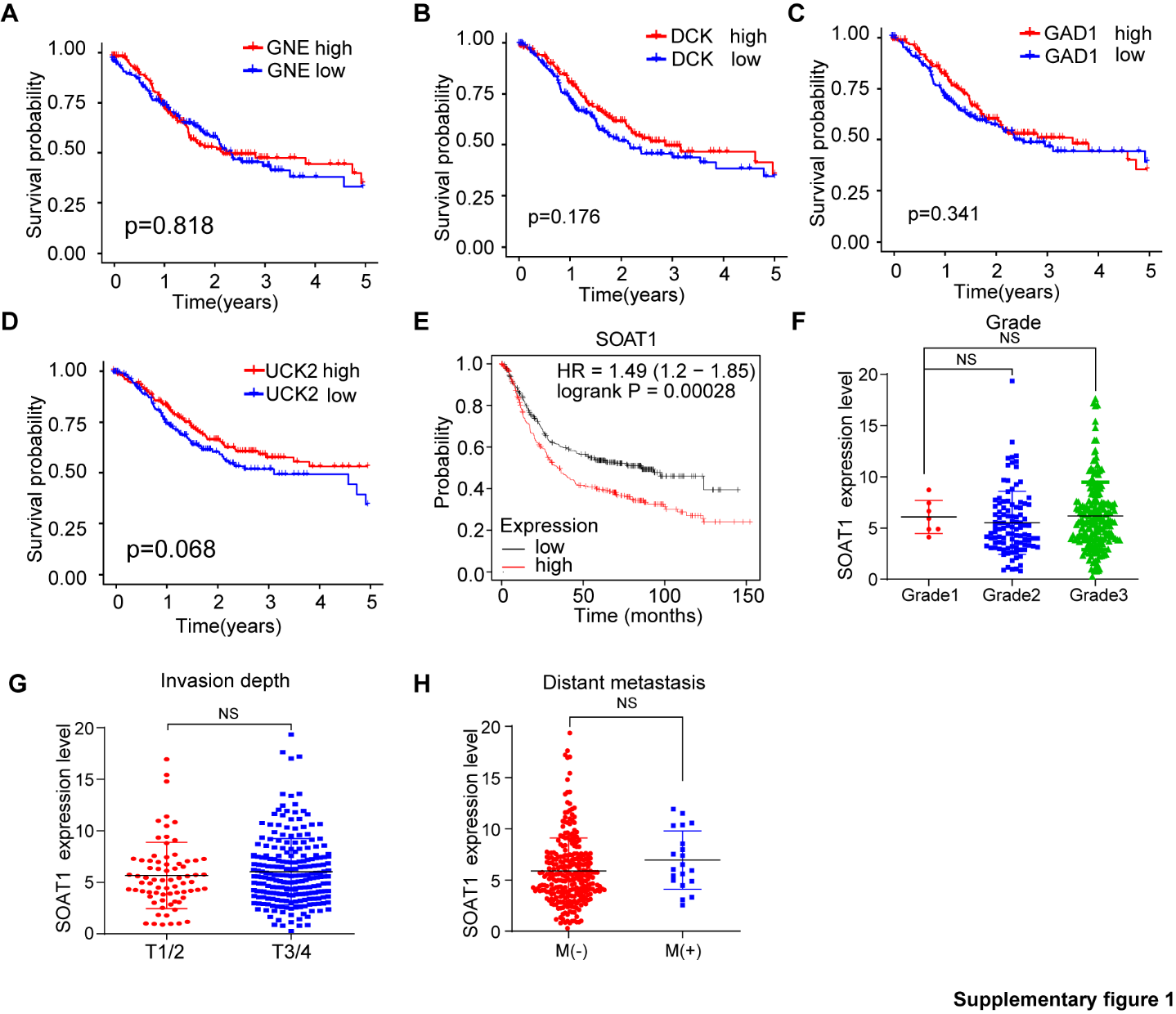


**Supplementary Figure 1 Selection of metabolic rate-limiting enzymes related to survival.**

(**A**-**D**) Kaplan–Meier curves showed overall survival according to the expression of GNE, DCK, GAD1, and UCK2. (E) Overall survival curves were plotted for all gastric adenocarcinoma patients with different levels of SOAT1 expression by Kaplan-Meier Plotter. (**F**-**H**) Correlation of SOAT1 expression with pathological grade (**F**), tumor invasion depth (**G**) and distant metastasis status (**H**).

**
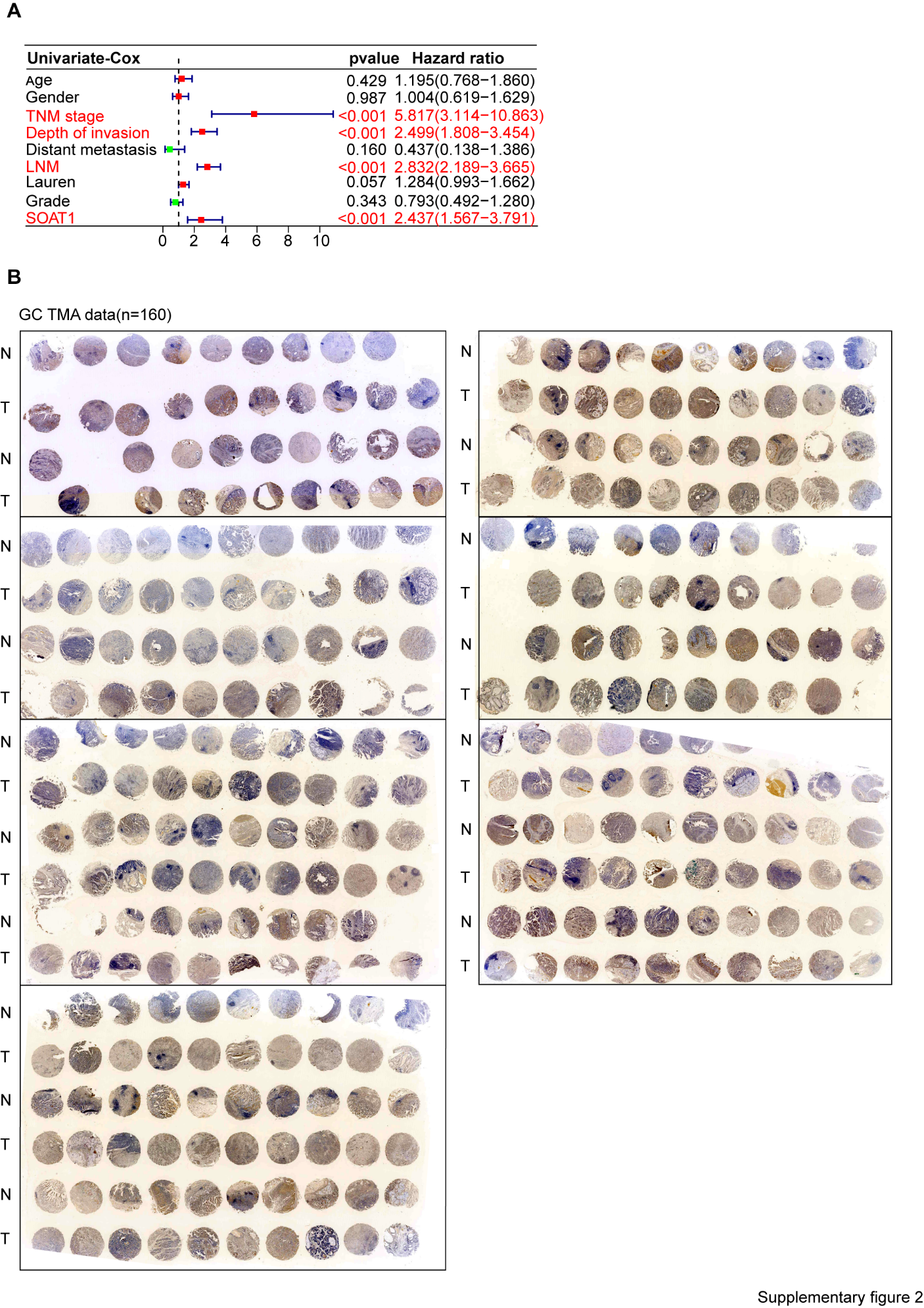
**

**Supplementary Figure 2** **Univariate analyses and the whole gastric cancer tissue microarray data**

**(A)** univariate analyses were performed with the above-mentioned GC patients, and all bars correspond to 95% confidence intervals (CIs) and hazard ratios (HRs). (B) the whole gastric cancer tissue microarray data


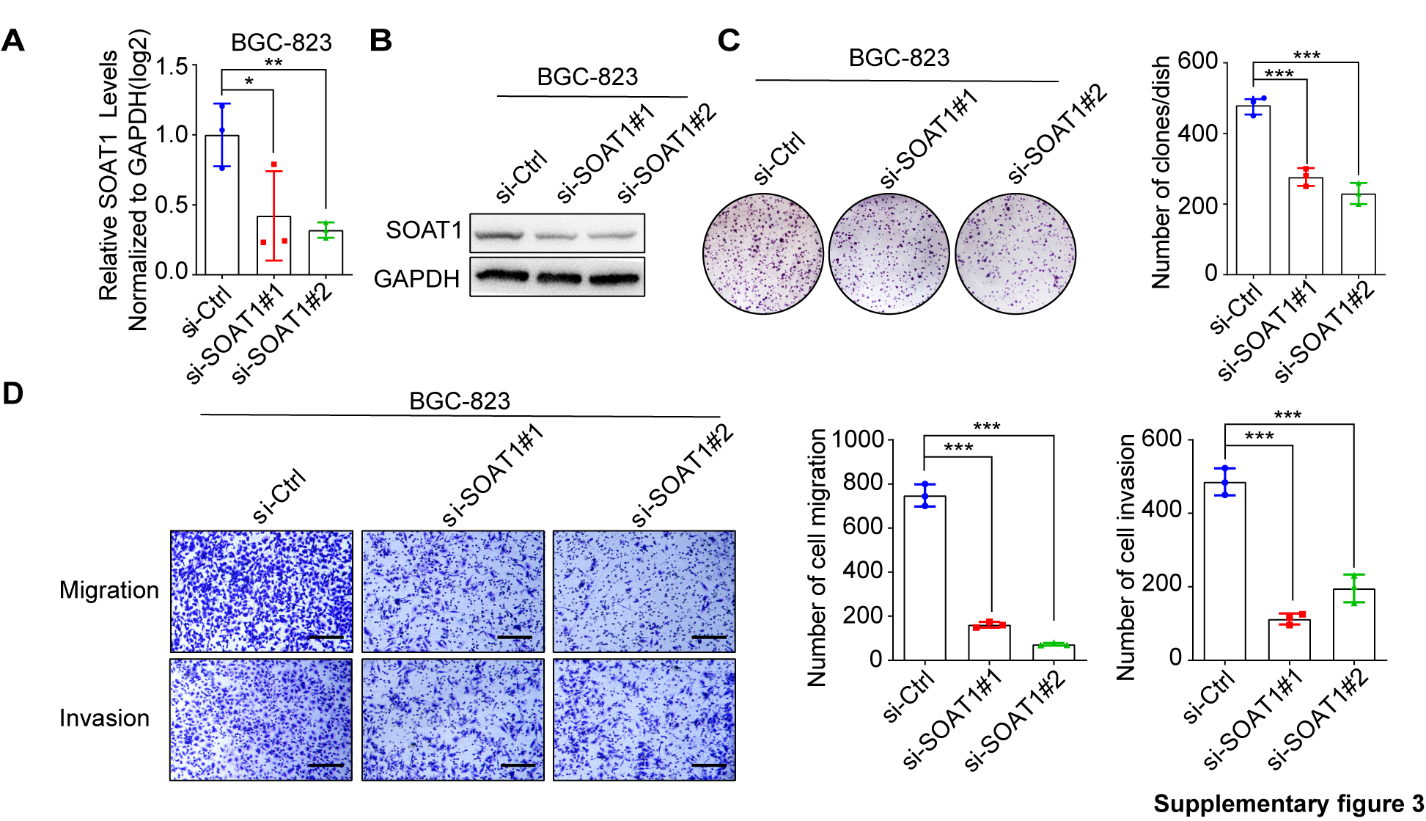


**Supplementary Figure 3 Knockdown SOAT1 suppresses GC cell growth and metastasis.**

(**A, B**) The knockdown efficiency was verified at the mRNA and protein levels by qRT-PCR (**A**) and western blot (**B**), respectively. (**C**) Representative images of colony formation assay in BGC-823 cells after Knockdown of SOAT1(le ft panel), the right panel shows the quantitative results. (**D**) Transwell assay in BGC-823 cells after knockdown of SOAT1. Representative images of migrated and invaded GC cells in each group were shown in the left panel, and the quantitative results were shown in the right panel. Three independent experiments were carried out. Scale bars: 100 µm


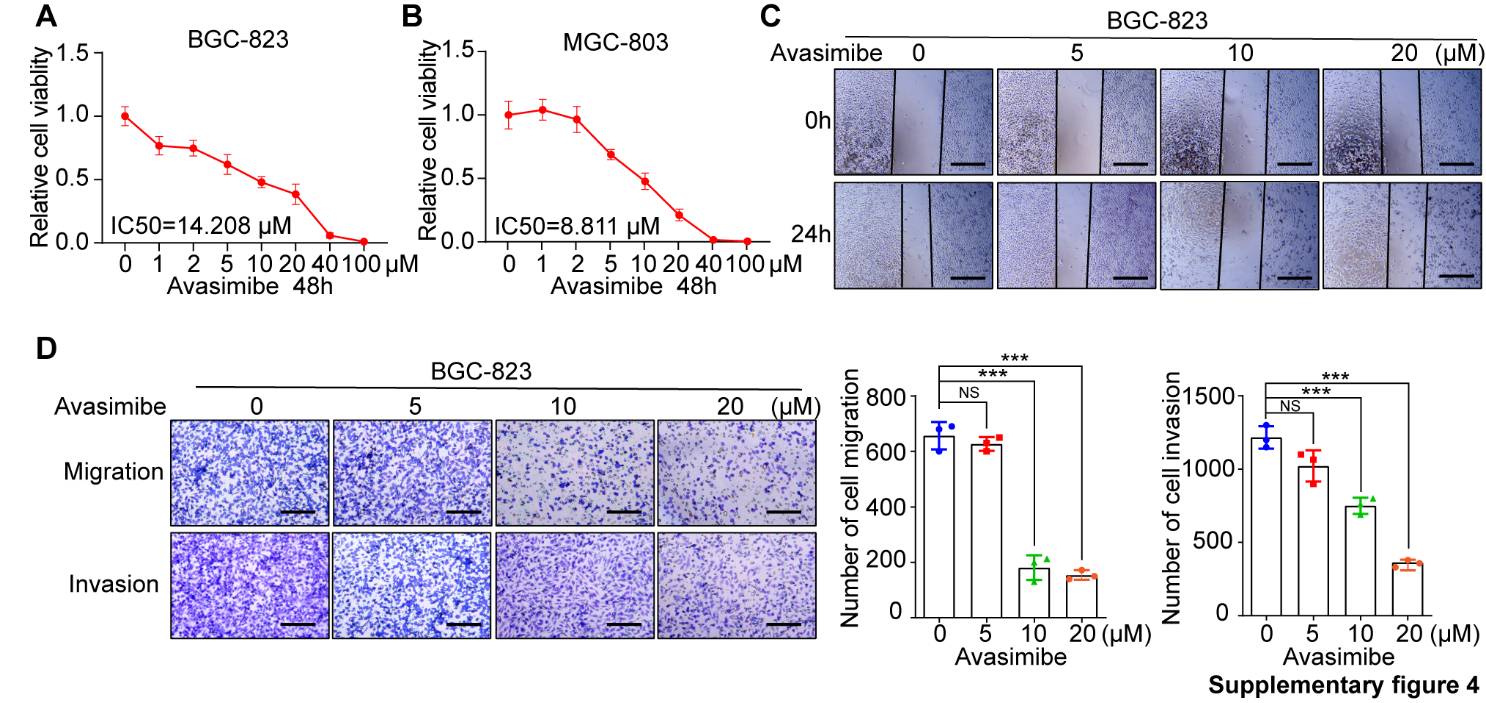


**Supplementary Figure 4 Avasimibe treatment decreased the metastasis capability of GC cells.**

(**A**, **B**) Relative cell viability was measured by CCK-8 assay after avasimibe treatment for 48 h at multiply concentrations (0, 1, 2, 5, 10, 20, 40, and 100 µM) in BGC-823 (**A**) and MGC-803 (**B**) cells. (**C**) Representative images of wound healing assay in BGC-823 cells showing cell motility after avasimibe treatment. (**D**) Transwell assay in BGC-823 cells after avasimibe treatment. Representative images of migrated and invaded GC cells in each group were shown in the left panel, and the quantitative results were shown in the right panel. Scale bars: 100 µm


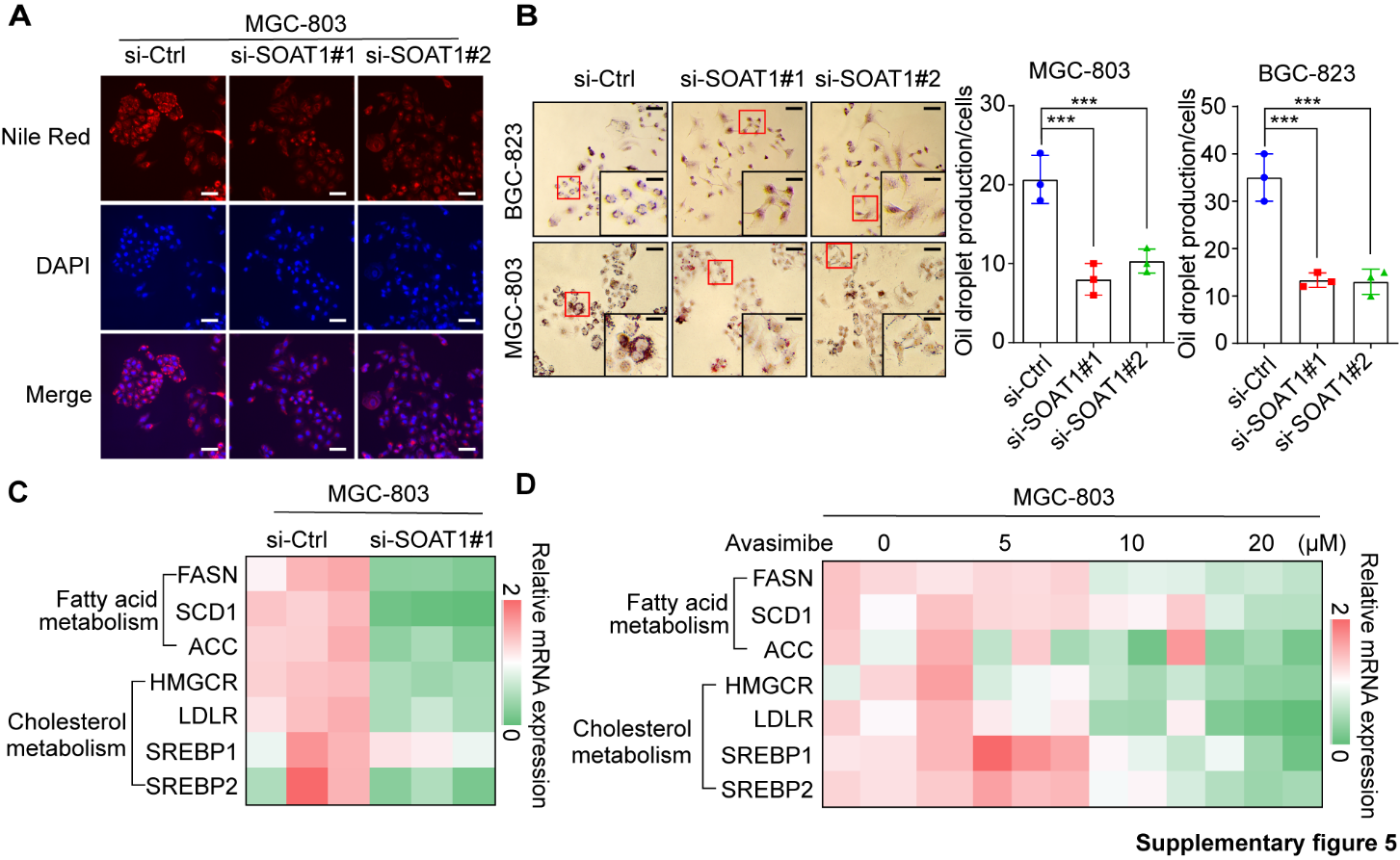


**Supplementary Figure 5 knockdown SOAT1 suppress GC cell lipid synthesis.**

(**A**) Representative images of Nile red stanning assay in MGC-803 cells after knockdown of SOAT1. Scale bars: 100 µm. (**B**) Representative images of Oil red O staining in BGC-823 and MGC-803 cells after knockdown of SOAT1 (upper panel); quantitative data for lipid synthesis were shown (lower panel). Scale bars: 100 µm and 50 µm, respectively. (**C**, **D**) Heatmap generated from the qRT-PCR results showed the gene expression levels of cholesterol metabolism and fatty acid biosynthesis-related genes in MGC-803 cells after knockdown of SOAT1 (**C**) or avasimibe treatment (**D**).


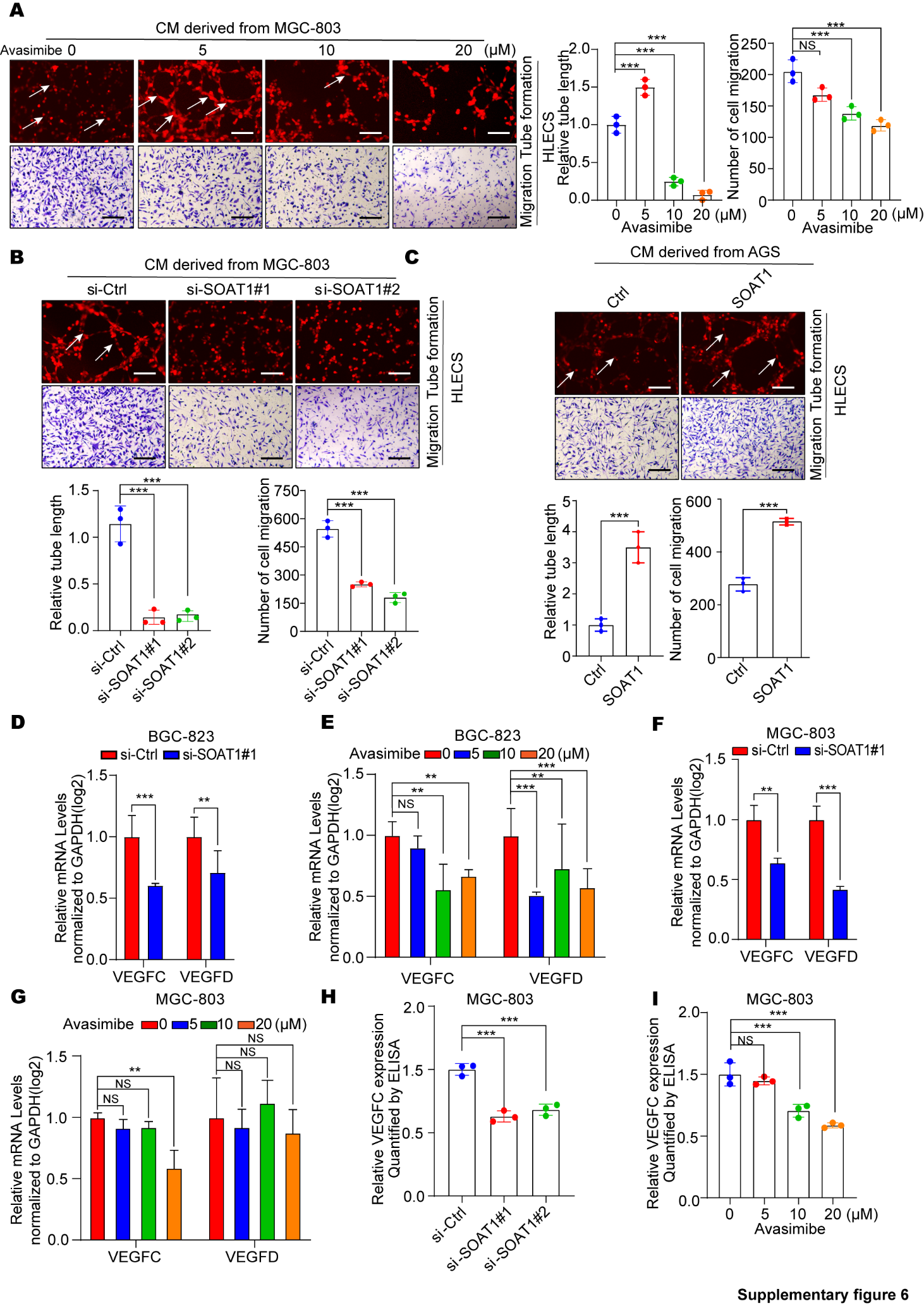


**Supplementary Figure 6 SOAT1 overexpression promotes lymphangiogenesis.**

(**A**) Representative images (upper panel) and quantitative analysis (lower panel) of tube formation and Transwell migration assays of HLECs cultured with conditioned medium collected from avasimibe-treated MGC-803 cells. (**B**, **C**) Representative images (upper panel) and quantitative analysis (lower panel) of tube formation and Transwell migration assay of HLECs cultured with conditioned medium collected from SOAT1-knockdown MGC-803 cells(**B**) or AGS-SOAT1 cells(**C**). Scale bars: 100 µm (**D-G**) The expression of VEGFC and VEGFD at the mRNA level were measured by qRT-PCR after knockdown of SOAT1 or avasimibe treatment in BGC-823 and MGC-803 cells. (**H-I**) ELISA assay of VEGFC expression in conditioned medium collected from the SOAT1 knockdown or avasimibe treated MGC-803 cells.

# Supplementary Tables

**Supplementary Table S1: The list of genes encoding 111 rate-limiting metabolic enzymes**

| ACO1 | ALDH7A1 | DPYD | HK2 | NAT1 | PYGM | TK1 |
| --- | --- | --- | --- | --- | --- | --- |
| ACO2 | ALDH9A1 | DTYMK | HK3 | NAT2 | RDH5 | TPH1 |
| ACSL1 | ALOX5 | F2 | HMGCR | OGDH | REN | TPH2 |
| ADH1A | APRT | FBP1 | HMGCS1 | OGDHL | RRM1 | TYMP |
| ADH7 | ASS1 | FBP2 | HMGCS2 | PAH | RRM2 | TYR |
| ADK | BACE1 | G6PC | HSD17B6 | PCK1 | RRM2B | UCK1 |
| ALAD | CHAT | G6PC2 | HSD3B1 | PCK2 | SAT1 | UCK2 |
| ALAS1 | COX4I1 | G6PD | HSD3B2 | PIK3C3 | SCD | UCKL1 |
| ALAS2 | COX5B | GAD1 | IMPDH1 | PKLR | SCD5 | UGDH |
| ALDH1A1 | COX6A1 | GAD2 | IMPDH2 | PKM2 | SOAT1 | UGT2B4 |
| ALDH1A2 | COX6A2 | GGT1 | LPCAT2 | PLAT | SOAT2 | XDH |
| ALDH1A3 | COX6B1 | GNE | LTA4H | PLAU | SPTLC1 |  |
| ALDH1B1 | COX6C | GPAM | LTC4S | PTGS1 | SPTLC2 |  |
| ALDH2 | DDC | GPD2 | MYLK | PTGS2 | SQLE |  |
| ALDH3A1 | DLD | HDC | MYLK2 | PYGB | STS |  |
| ALDH3A2 | DLST | HK1 | MYLK3 | PYGL | TAT |  |

# Supplementary Table S2: Relationship between expression level of SOAT1 and demographic and clinicopathological features of GC patients.

| Variables | SOAT1 expression | |  |
| --- | --- | --- | --- |
|  | Low (%) | High (%) | p-value |
| All patients | 109(68.1) | 51(31.9) |  |
| Age, years, mean ± SD | 62.31±11.322 | 62.37±9.284 | 0.973 |
| Gender |  |  | 0.533 |
| Male | 76(69.7) | 38(74.5) |  |
| Females | 33(30.3) | 13(25.5) |  |
| Depth of invasion |  |  | 0.195 |
| T1/T2 | 27(24.8) | 8(15.7) |  |
| T3/T4 | 82(75.2) | 43(84.3) |  |
| Lymph node metastasis |  |  | 0.001 |
| N0 | 33(30.3) | 9(17.6) |  |
| N1 | 27(24.8) | 3(5.9) |  |
| N2 | 17(15.6) | 11(21.6) |  |
| N3 | 32(29.4) | 28(54.9) |  |
| Distant metastasis |  |  | 0.328 |
| M0 | 107(98.2) | 48(94.1) |  |
| M1 | 2(1.8) | 3(5.9) |  |
| TNM stage |  |  | 0.029 |
| Ⅰ/Ⅱ | 45(41.3) | 12(23.5) |  |
| Ⅲ/Ⅳ | 64(58.7) | 39(76.5) |  |
| Tumor diameter(cm) |  |  | 0.697 |
| ≤5 | 69(63.3) | 34(66.7) |  |
| ＞5 | 40(36.7) | 17(33.3) |  |
| Histological type |  |  | 0.366 |
| Intestinal | 53(48.6) | 20(39.2) |  |
| Diffuse | 29(26.6) | 19(37.3) |  |
| Mixed | 27(24.8) | 12(23.5) |  |
| Differentiation degree |  |  | 0.006 |
| Differentiated | 43(39.4) | 9(17.6) |  |
| Undifferentiated | 66(60.6) | 42(82.4) |  |

# Supplementary Table S3：The primer sequences for qRT-PCR.

| Primer names | Sequences (5’-3’) |
| --- | --- |
| For qRT-PCR |  |
| GAPDH F | CTGGGCTACACTGAGCACC |
| GAPDH R | AAGTGGTCGTTGAGGGCAATG |
| SOAT1 F | GAAGTTGGCAGTCACTTTGATGA |
| SOAT1 R | GAGCGCACCCACCATTATCTA |
| SREBP1 F | ACAGTGACTTCCCTGGCCTAT |
| SREBP1 R | GCATGGACGGGTACATCTTCAA |
| SREBP2 F | CCTGGGAGACATCGACGAGAT |
| SREBP2 R | TGAATGACCGTTGCACTGAAG |
| LDLR F | TCTGCAACATGGCTAGAGACT |
| LDLR R | TCCAAGCATTCGTTGGTCCC |
| HMGCR F | TGATTGACCTTTCCAGAGCAAG |
| HMGCR R | CTAAAATTGCCATTCCACGAGC |
| ACC F | ATGTCTGGCTTGCACCTAGTA |
| ACC R | CCCCAAAGCGAGTAACAAATTCT |
| SCD1 F | TCTAGCTCCTATACCACCACCA |
| SCD1 R | TCGTCTCCAACTTATCTCCTCC |
| FASN F | AAGGACCTGTCTAGGTTTGATGC |
| FASN R | TGGCTTCATAGGTGACTTCCA |
| VEGF-C F | GAGGAGCAGTTACGGTCTGTG |
| VEGF-C R | TCCTTTCCTTAGCTGACACTTGT |
| VEGF-D F | ATGGACCAGTGAAGCGATCAT |
| VEGF-D R | GTTCCTCCAAACTAGAAGCAGC |

# Abbreviations: F, Forward; R, Reverse
